# Transplantation of Adult Monkey Neural Stem Cells into A Contusion Spinal Cord Injury Model in Rhesus Macaque Monkeys

**Published:** 2014-05-25

**Authors:** Shiva Nemati Nemati, Reza Jabbari, Mostafa Hajinasrollah, Nargess Zare Mehrjerdi, Hossein Azizi, Katayoun Hemmesi, Reza Moghiminasr, Zahra Azhdari, Ardeshir Talebi, Soroush Mohitmafi, Ahmad Vosough Taqi Dizaj, Giuve Sharifi, Hossein Baharvand, Omidvar Rezaee, Sahar Kiani

**Affiliations:** 1 Department of Stem Cells and Developmental Biology at Cell Science Research Center, Royan Institute for Stem Cell Biology and Technology, ACECR, Tehran, Iran; 2 Department of Neurosurgical Science, Loghman Hospital, Shahid Behshti University of Medial Sciences, Tehran, Iran; 3 Al-Zahra Hospital, Isfahan University of Medical Sciences, Isfahan, Iran; 4 Department of Clinical Science, Faculty of Veterinary Medicine, Karaj Branch, Islamic Azad University, Karaj, Iran; 5 Department of Reproductive Imaging at Reproductive Biomedicine Research Center, Royan Institute for Reproductive Biomedicine, ACECR, Tehran, Iran; 6 Department of Developmental Biology, University of Science and Culture, ACECR, Tehran, Iran

**Keywords:** Neural Stem Cell, Spinal Cord Injury, Primates, Transplantation

## Abstract

**Objective:**

Currently, cellular transplantation for spinal cord injuries (SCI) is the subject of numerous preclinical studies. Among the many cell types in the adult brain, there is a unique subpopulation of neural stem cells (NSC) that can self-renew and differentiate into neurons. The study aims, therefore, to explore the efficacy of adult monkey NSC (mNSC) in a primate SCI model.

**Materials and Methods:**

In this experimental study, isolated mNSCs were analyzed by flow cytometry, immunocytochemistry, and RT-PCR. Next, BrdU-labeled cells were transplanted into a SCI model. The SCI animal model was confirmed by magnetic resonance imaging (MRI) and histological analysis. Animals were clinically observed for 6 months.

**Results:**

Analysis confirmed homing of mNSCs into the injury site. Transplanted cells expressed neuronal markers (TubIII). Hind limb performance improved in trans- planted animals based on Tarlov’s scale and our established behavioral tests for monkeys.

**Conclusion:**

Our findings have indicated that mNSCs can facilitate recovery in contusion SCI models in rhesus macaque monkeys. Additional studies are necessary to determine the im- provement mechanisms after cell transplantation.

## Introduction

Recovery of adult mammals after spinal cord injuries (SCI), which are the most common of central nervous system (CNS) complications in humans, is hindered by the limited ability of the CNS to replace lost cells, axonal growth inhibitors associated with myelin and glial scars, and insufficient trophic support (1).

Due to recent progress in stem cell biology, cellular transplantation for treating SCI has been the subject of numerous preclinical studies. Various cell types have been used based on their potential to form myelin, promote and guide axonal growth and bridge the injury site. In addition, trophic factors which are secreted from transplanted cells may have neuro-protective effects and/or promote plasticity in the spared spinal cord. However, the privilege of these kinds of therapies are multi-factorial and often difficult to refer to one single mechanism such as transplanted cell types and potencies (2). Several therapeutic strategies have been developed to manipulate these molecules in an attempt to promote axonal growth and replace lost neurons after SCI.

There are many types of stem cells which can be used for cell therapy purposes. However, the ideal "transplantable cells" should be accessible, rapidly expandable in culture, immunologically inert, capable of long-term survival and integration in the host tissue, and amenable to stable transfection and expression of exogenous genes. In the adult brain, there is a unique subpopulation of neural stem cells (NSCs) that have the capability to self-renew and differentiate into neurons and glia (3).

In recent years researchers have focused on these types of NSCs to achieve their therapeutic goals. The mentioned cells proliferate as floating clusters which are introduced as neurospheres in the presence of some growth factors such as epidermal growth factor (EGF) or basic fibroblast growth factor (bFGF) (4, 5).

Most previous SCI model studies have been conducted in rodents (6-8). The results of these studies are not directly applicable to patients with SCI because of the differences in neuro- functional and anatomic features between rodents and humans. For example, the corticospinal tract (CST) is located in the posterior funiculus of rodents; in humans, a major portion of this structure is present in the lateral funiculus with the remainder in the anterior funiculus (9, 10). In monkeys the CST is also present in the lateral funiculus. Research using the monkey as an SCI model will enable more accurate results for humans. Animal models using macaques and common marmosets (Callithrix jacchus) have already been established for diseases such as Parkinson’s (11, 12), SCI (1), and multiple sclerosis (13), contributing greatly to advancing research on these conditions.

Models for SCI research most frequently involve surgical exposure of the spinal cord. The impact model and its variants, first described by Allen, involve the dropping of a predetermined weight from a predetermined height onto an exposed spinal cord, thus producing a more precise quantification and standardization of the injury (1, 14, 15). This technique leads to a known trauma and has the advantage of producing a "shock" injury to the spinal cord.

This study aims to explore the efficacy of the safe and effective use of adult monkey NSCs (mNSCs) for the treatment of acute SCI in the rhesus monkey.

## Materials and Methods

### Experimental animals

In this experimental study, we used six normal rhesus (Maacca mulatta) monkeys (ages: 3-6 years old; weight: 3-6 kg) for these experiments. This experiment was approved by the Ethical Committee at Royan Institute. The animals were gifted from the Primate Research Center of Royan Institute. The monkeys were seronegative for tuberculosis, Simian immunodeficiency virus (SIV), herpes B, hepatitis A and B viruses, and free from intestinal parasites. Animals were randomly divided into 2 groups: experimental (n=4) and control (n=2).

### Isolation and culture of mNSCs

We generated 2 adult mNSC cultures from the brains of 2 adult monkeys. The monkeys were followed for other studies at Royan Institute and euthanized for histological analyses. For each culture, we used identical dissection, dissociation, and culture protocols. Briefly, a small piece of the sub-ventricular zone (SVZ) from the adult monkey brain was isolated and refrigerated overnight in preservative medium that consisted of Dulbecco’s modified Eagle medium (DMEM) F12 (Invitrogen, Grand Island, USA), gentamicin (Sigma-Aldrich, Saint Louis, USA), and amphotericin (Invitrogen, Scotland).

Next, dissected tissues were enzymatically dissociated in 0.8 mg/ml hyaluronidase and 0.5 mg/ml trypsin in DMEM that contained 4.5 mg/ml glucose (Invitrogen, Grand Island, USA) and 80 U/ml DNase (Sigma-Aldrich, Saint Louis, USA) at 37˚C for 20 minutes. Cells were gently mixed with 3 volumes of NSC expansion medium. Expansion medium consisted of DMEM/F12, 2% B27 supplement, 10% FBS, NEAA, L-glutamine, 100 U/ ml penicillin, and 100 μg/ml streptomycin (all from Invitrogen), 20 ng/ml EGF and bFGF (Sigma-Aldrich). After filtering through a 70 μm strainer, cells were pelleted at 160×g for 5 minutes. The supernatant was subsequently removed and cells resuspended in neurosphere medium supplemented as above, plated in uncoated culture dishes, and incubated at 37˚C. After 4 days, the supernatant was replaced with fresh expansion medium. The medium was renewed every other day. At 8-10 days after plating, the primary culture was ready to be passaged. Other passages were performed weekly; cells were available for use after at least 10 passages.

### Characterization of monkey neural stem cells

The expressions of NSC markers on mNSCs were assessed by flow cytometry (Beckman Coulter, Miami, FL) as previously described (16). mNSCs were detached by trypsinization, then stained with specific antibodies to nestin (1:100, Chemicon, MAB5326), Sox1 (1:1000, Abcam, Cambridge, UK, 22572), and Pax6 (1:100, Santa Cruz, CA, USA, 11357) in addition to secondary fluorescent antibodies, FITC anti-mouse IgG (1:200, Chemicon, AP308F) and FITC anti- rabbit IgG (1:200, Sigma-Aldrich, F1262).

To visualize alkaline phosphatase (AP) activity (17), isolated mNSCs were fixed and stained by the BCIP/NBT Phosphatase Substrate System (KLP, Gaithersburg, MD) following the manufacturer’s instructions and observed with a light microscope. Spontaneous differentiation was performed in differentiation medium in the absence of growth factors, which included neurobasal medium, B27 (1%), FBS (10%), and N2 supplement (1%) for 35 days. Half of the medium was renewed every 5 days. To characterize neuronal differentiation we used immunofluorescence staining (15) for TUJ1 (1:400, Sigma-Aldrich, T8660), MAP2 (1:400, Sigma-Aldrich, M1406), and GFAP (1:400, Sigma-Aldrich, G3893). Cell nuclei were counterstained with 4, 6-diamidino-2-phenylindole (DAPI).

For each sample, total RNA was extracted using the RNX reagent (Cinnagen, RN7713C, Tehran, Iran). A total of 5 μg RNA was treated with DNase1 (Fermentas, Vilnius, Lithuania). cDNA was synthesized from 1 μg of RNA using the Revert Aid TM H Minus First Strand cDNA Synthesis Kit (Fermentas, Vilnius, Lithuania) with a random hexamer primer. The resulting cDNA were subjected to RT-PCR. Specific human primers MAP2, β-tubulin III, Pax6, and Sox1 (Table 1) were designed with Perl primer V.1.1.14 software. For each PCR run, 50 ng cDNA products were mixed with 1.5 mM MgCl2, 0.2 mM dNTP mix, 1X PCR buffer (Cinnagen, Iran), 0.2 pM each of antisense and sense primers, and 1U Taq DNA polymerase. PCR reactions were performed on a Master cycler gradient machine (Eppendorf, Germany). The reaction was carried out at 94˚C for 4 minutes, followed by 94˚C for 45 seconds, an annealing temperature for 45 seconds, and 72˚C for 45 seconds for a total of 35 cycles, followed by a final extension at 72˚C for 10 minutes. The PCR product was electrophoresed through a 1.7% tris acetate-EDTA buffer (TAE) agarose gel. The gels were stained with 0.5 μg/ ml ethidium bromide and visualized on a UV transilluminator (UVIdoc, UK).

**Table 1:** Primers for conventional reverse transcription-polymerase chain reaction


Genes	Primer sequences (5´-3´)	Accession no.

**Pax6**	F: CGGTTTCCTCCTTCACAT R: ATCATAACTCCGCCCATT	NM_000280
**Sox1 **	F: CCTCCGTCCATCCTCTG R: AAAGCATCAAACAACCTCAAG	NM_005986
**TUJ1**	F: GTATCCCGACCGCATCAT R: TCTCATCCGTGTTCTCCA	NM_006086
**MAP2**	F: TGAAGAACATCCGCCACA R: CTTGACATTACCACCTCCAG	NM_002374
**ActB**	F: TCCCTGGAGAAGAGCTACG R: GTAGTTTCGTGGATGCCACA	NM_ 001101.3


### Contusive spinal cord injury

**Fig 1:**
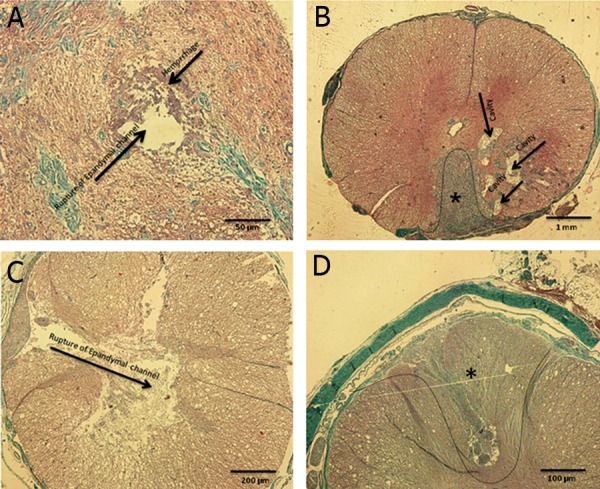
Histological assessment of rhesus monkey contusion model. A. Rupture of ependymal channel and hemorrhage. B, C. Cavity and fibrosis formation, severity of impact causes cavity deformation, D. Ependymal channel was damaged because of severity of impact and fibrosis formation (green region) as shown by Masson trichrome staining. *; Fibrosis formation.

**Fig 2:**
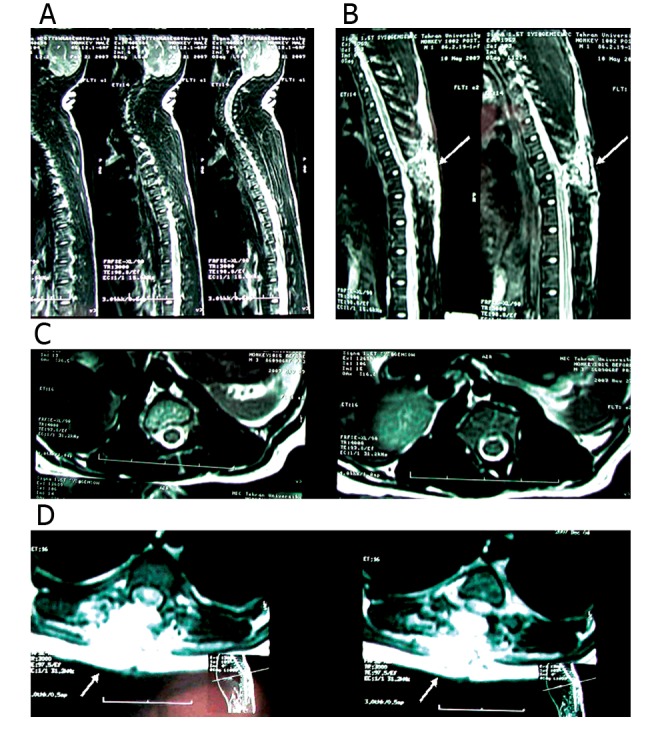
Illustration of the selected regions of interest. A. Sagittal section of spinal cord region of the corrected phase images. B. Sagittal section of region of the injured spinal cord phase images, C. Cross-section of spinal cord region of the corrected phase images, D. Cross-section of injured spinal cord region of the phase images. B, D. The pointers show the surgical effects of the midline incision on the course of events at the contusion site. These abnormally elevated signal intensities were noted at the T9-11 level which were compatible with cord edema and confirmed the contusion injury.

In this study contusive SCIs were induced in rhesus monkeys using a modified NYU device according to a modified Allen’s method (18). After an intramuscular (im) injection of atropine (0.25 mg/kg), anesthesia was induced by injection of ketamine (15 mg/kg, im) and xylazine (0.4 mg/kg, im). Laminectomy was performed on the thoracic vertebrate (T9- 10) and a 50 g weight dropped from a height of 12 cm through a guide tube onto a 10 mm^2^ impact plate over the exposed spinal cord, which caused trauma (19). All animals received twice daily cefazolin (Fig 1A-D) (25 mg/kg, im) during the first week. Manual bladder expression was performed at least 3 times each day until voluntary urination was established. Paralyzed animals were given adequate amounts of food and water until they recovered their ability to eat and drink without assistance. Magnetic resonance imaging (MRI) of the injured spinal cord was conducted 1 week before and 2 days after injury as follows: i. sagittal and ii. axial T1-weighted, and T2-weighted images between C5-L1 (Fig 2A-D) using a 1.5-Tesla superconducting imager (Sigma, Milwaukee, WI) with a phased-array volume coil. Images proved SCI by modified Allen’s method and induced our contusive model. The magnitude of the SCI was monitored by examining changes in intramedullary MRI signals. Changes such as hemorrhage, edema, and cavity formation can be monitored in real time using MRI (20, 21).

### Histological procedure

After 6 months, we euthanized one control animal for histological analysis. Paraffin blocks of the traumatized area (zone) and the upper and lower zones of the injured spinal cord were prepared and stained for collagen, reticulin, and elastin fibers using Masson’s trichrome and Verhoeff’s staining procedure. In addition, we prepared kidney, liver and heart tissue paraffin blocks for analysis of tumorigenesis. Immunohistochemistry was performed as previously described (15).

### Transplant procedure

**Fig 3:**
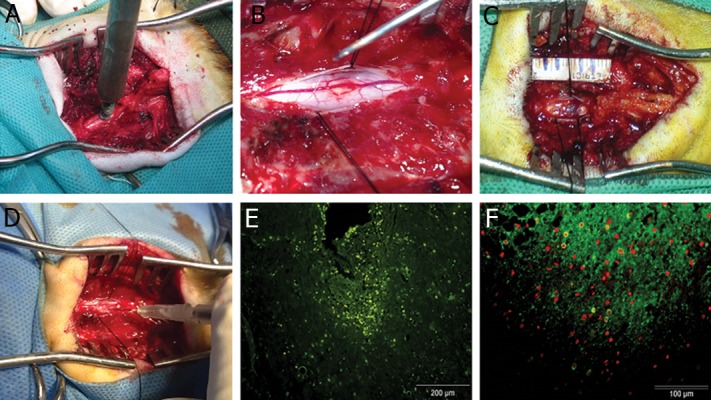
Schematic illustration of surgery and cell transplantation procedure. A. Modeling procedure and dropping a 50 g weight over the exposed cord. B, C. Dissection of dura matter, site and length of injured cord. D. Cells were injected throw at least 10 region in the site of injury. E. Immunohistochemistry staining showed homing of BrdU positive transplanted cells into the injury site of the spinal cord. F. Immunohistochemistry staining for Tuj1 positive cells. *; Fibrosis formation.

The transplant was performed in a randomized, blind design 10 days post-injury [all animals: grade 0 [according to Tarlov’s scale (22)] at which time the microenvironment of the injured spinal cord changed from the acute phase to one that supported the survival and differentiation of transplanted cells. All procedures were performed within 10 days as to minimize differences in protocol between experimental and control animals. In the experimental group (n=4) isolated, BrdU-labeled mNSCs (1×10^6^ cells/kg) were directly transplanted into the traumatized area. mNSCs were trypsinized at passage 11 and washed with PBS buffer prior to transplantation. The control group (n=2) underwent no treatment (Fig 3A-C). Ceftriaxon was administered daily to each animal at a dose of 30 mg/kg (im). Animals received daily injections of cyclosporin (5 mg/kg, im, Novartis, Basel, Switzerland) for 6 months following transplantation.

### Behavioral evaluation

Before and 3 days after each surgical procedure or transplantation, 2 neurosurgeons blinded to the protocol performed behavior analysis on each of the animals. Behavior analysis was also conducted weekly following transplantation for 6 months.

### Measurement of spontaneous motor activity

Previous studies of SCI in rodents have utilized 2-dimensional (2D) functional evaluation (23-25). Most rhesus monkeys are 3-dimensional (3D) in that they jump and climb cages; therefore measurement of spontaneous motor activity is difficult. For this reason, we have evaluated motor activity by assessments with Tarlov’s scale (22) and tail movements.

### Tarlov’s scale

In the first intervention, we graded each animal’s neurologic function according to the modified Tarlov et al. scale (22), as follows: grade 0 (no voluntary function); grade 1 (perceptible joint movement); grade 2 (active joint movement, but the animal is unable to stand); grade 3 (animal is able to stand but unable to hop); and grade 4 (complete recovery with no neurologic deficit) (26, 27).

### Tail movements

We divided animal tail movements into 4 parts: 0 (no movement); 1 (weak movement); 2 (powerful movement); and 3 (voluntary reflex).

### Measurement of stimulatory motor activity

Stimulator motor activity was measured by the tail pinch and lower limb pinch tests. These tests were established by our group to evaluate the grade of SCI for the first time; accordingly, the hind limb force was also evaluated.

### Limb pinch test

The limb pinch test involved pinching the hind limb toe with forceps. Reflex action to the limb pinch was divided into 4 grades: 0 (no reflex); 1 (weak reflex); 2 (powerful reflex); and 3 (voluntary reflex).

### Tail pinch test

The top of the tail was pinched with forceps and the animal’s reaction was divided into 4 grades: 0 (no reflex); 1 (weak reflex); 2 (powerful reflex); and 3 (voluntary reflex).

### Sensory tests

Sensory tests were performed by stimulating the animal’s hind limb by a controlled brief pinch (needle) to observe the pain withdrawal reflex. This test was performed weekly and graded according to the pain reaction as grades 0 (no reflex) and 1 (voluntary reflex). We additionally performed Babinski and bulbocavernosus tests on the animals.

### Statistical analysis

All numerical data are presented as mean ± SEM. Parametric tests (one-way ANOVA with subsequent Tukey’s post hoc tests) were used for comparisons as between groups. Paired student’s t test was applied to compare the differences in both groups. Analyses were performed using software package SPSS 16 and the level of significances was p<0.05.

## Results

### Isolation and purification and characterization of monkey neural stem cells

**Fig 4:**
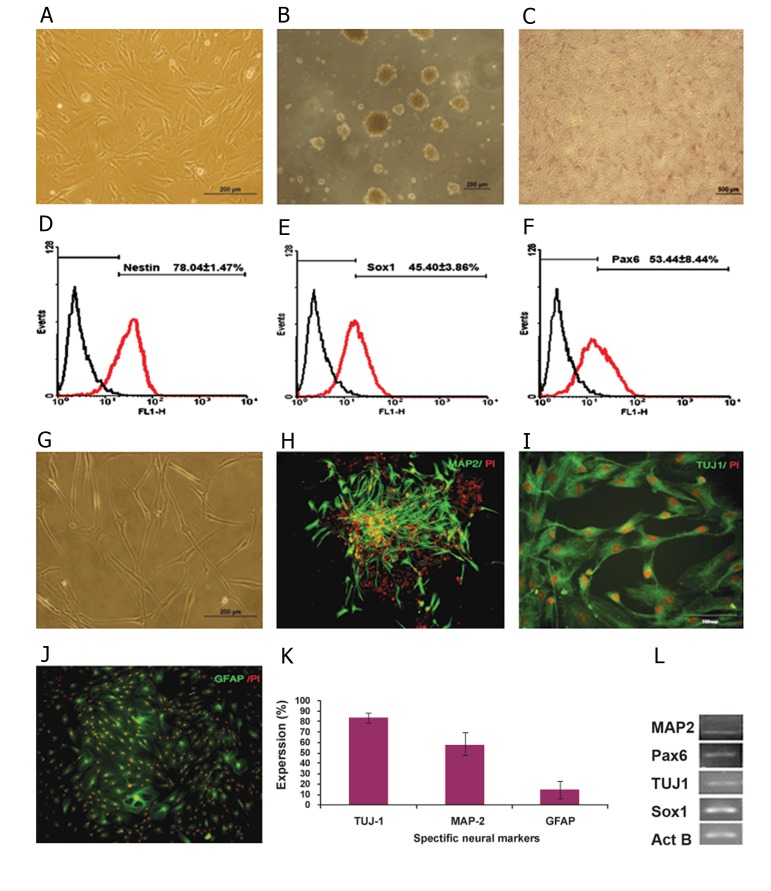
Monkey neural stem cell (mNSCs) culture and characterization at passage 11. A. Confluent mNSCs isolated from the sub-ventricular zone (SVZ). B. Passageable neurosphere formation from mNSCs. C. Alkaline phosphatase (AP) staining for mNSCs. D-F. Flow cytometry analysis for nestin, Sox1 and Pax6 expression for mNSCs. G. Phase contrast spontaneous differentiation of mNSCs after 14 days. H-J. Immunoflurescent staining for neural differentiated markers. K. Quantification of differentiated mNSCs, L. PCR analysis of mNSCs for neural markers.

Monkey NSCs were isolated from tissue extract by their adherence to the culture dishes. After primary culture for 5 days, we observed a morphologically homogeneous population with spindle shaped cells. The cloned cells were observed at days 6-10. Primary cultures reached 85% confluency in an expansion medium at 12 days (Fig 4A). Since one of the main features of mNSCs is their ability to form passageable neurospheres, we have analyzed this property on our isolated mNSCs by culturing neurospheres derived from mNSCs in the presence of expansion media (Fig 4B).

mNSCs were positive for AP, a prototypical marker for embryonic stem (ES) cells (Fig 4C). Phenotypic analysis of mNSCs expanded in culture during passages 10-15 was studied by flow cytometry which indicated that our isolated cells were positive for NSC markers (Fig 4D-F) nestin (78.04 ± 1.47%), Sox1 (45.50 ± 3.86%), and Pax6 (53.44 ± 8.44%). To assess the differentiation potential of mNSCs, cells were differentiated by growth factor withdrawal. After 35 days, mNSCs spontaneously differentiated into neurons and astrocytes, a property consistent with normal multipotent mNSCs (Fig 4G). The differentiated cells expressed neural specific markers Tuj1, MAP2, and GFAP for astrocytes (Fig 4H-J).

The expression of NSC transcription factors in mNSCs was studied by RT-PCR, which detected expressions of Map2, Pax6, Sox1, and Tuj1 (Fig 4K).

### MRI evaluation

MRI is currently the most powerful clinical tool available for the detection of pathologic events *in vivo* (28). Although MRI is far less sensitive than histology, the effects of microsurgery can be monitored by *in vivo* MRI, further confirming histological data.

Spinal cord MRI of the lesion showed the surgical effects of the midline incision on the course of events at the contusion site. These effects were observed on the MRI images by a comparison between the anatomy of the injured cords and the normal cord (Fig 2A-D). In the injured cords, a high signal area at the T1W sequence compatible with hemorrhagic contusion was noted at the laminectomy (T10-11) site. In addition, an abnormally elevated signal intensity was noted at the T9-11 level compatible with cord edema (cord expansion was not seen). Localized CSF accumulation was also noted at the laminectomy site without compression or thecal sac (29).

### Histological analysis

Histochemical analysis identified the background matrix as strongly positive for collagen per Masson’s trichrome and Verhoeff’s staining, which indicated the presence of fibrosis. All sections stained negative for reticulin and elastin. Immunofluorescent staining that traced transplanted cells showed the presence of previously labeled BrdU-positive cells which had been labeled prior to transplantation into the spinal cord. Also noted were a number of Tuj1-positive cells among the transplanted cells at the injured site (Fig 3E-F).

### Behavioral evaluation

**Fig 5:**
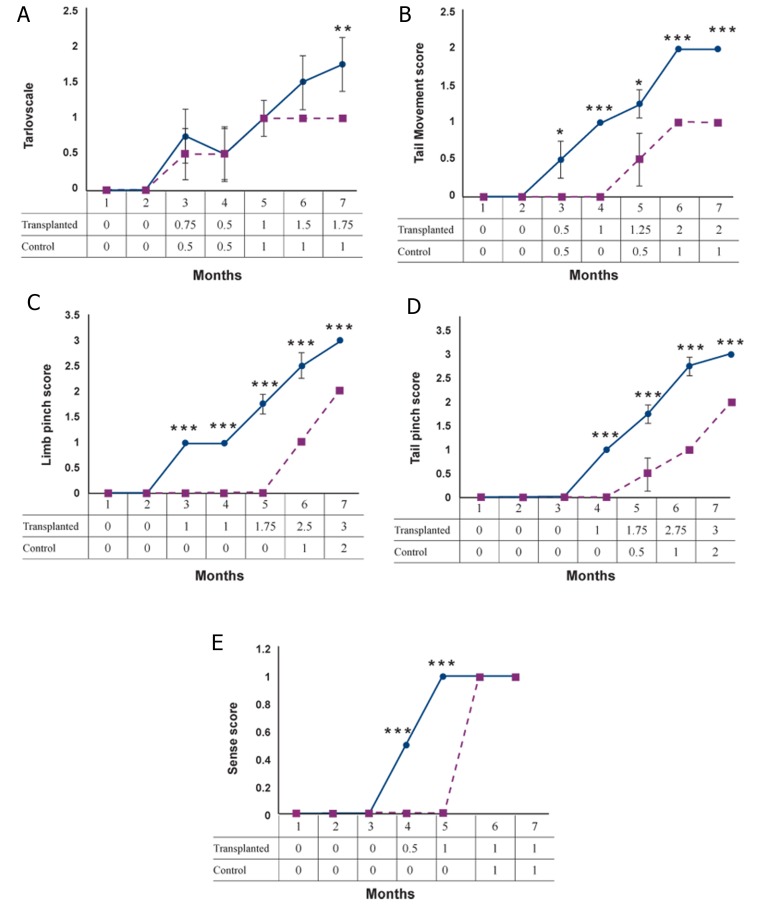
Behavior analysis was conducted weekly following transplantation for 7 months. A. Tarlov’s scale. B. Tail movements. C. Limb pinch test. D. Tail pinch test. E. Sensory tests. One-way ANOVA test was used for comparing data between both groups. Significance level: p<0.05; ***; p<0.001, **; p<0.01 and *; p<0.05.

One day after transplantation, 2 neurosurgeons blinded to the study groups began clinical observations of the monkeys which were performed twice weekly for up to 6 months. Approximately 10 days after transplantation, both experimental and control groups began to recover sensory responses. The normal pain withdrawal reflex was elicited by a controlled brief pinch of the tail and lower limbs, along with other sensory tests as performed by one of the neurosurgeons. In the transplanted and control groups there were significantly progressive trends in movement recovery and Tarlov’s scale during 7 months (paired t test, p<0.001). However a comparison of data between both groups showed that only in the last week of the study Tarlov’s scale in the transplanted group was significantly greater than that of the control group (one-way ANOVA, p<0.01, Fig 5A).

Tail movement score data showed significant differences in the transplanted group after the second month, however in the control group tail movement improved significantly in the forth last months. (Fig 5B). In both groups, there were no significant differences in last two weeks (paired t test, p<0.001). A comparison of tail movement data between the two groups showed that after the third month tail movement recovery was faster in the transplanted group compared to the control group (one-way ANOVA, p<0.001, Fig 5B).

In the transplanted group the limb pinch score significantly increased after the second month, but in the control group this increased trend began from the fourth month of the experiment (paired t test, p<0.001). Limb pinch scores in the transplanted group were significantly greater than seen in control animals (one-way ANOVA, p<0.001, Fig 5C).

Tail pinch and limb pinch scores showed similar significances. Tail pinch scores in both groups were significantly greater after the third month (paired t test, p<0.001); after the third month reflex action to the tail pinch in the transplanted group was significantly greater than in the control animals (one-way ANOVA, p<0.001, Fig 5D).

Sensory improvement showed a similar trend in both groups (paired t test, p<0.001) but in transplanted animals sensory functions improved faster than the control group (one-way ANOVA, p<0.001, Fig 5E).

The results of the bulbocavernosus test were the same as those seen after acute human SCIs, whereas the Babinski test was neutral in all cases both before and following SCI.

## Discussion

SCI is a traumatic complication responsible for a wide range of functional deficits. After the initial insult to the spinal cord, additional structure and function are lost through an active and complex secondary phase. Unfortunately no effective treatment has been introduced for SCI. A number of strategies that include cellular, pharmacological, and rehabilitation therapies have been utilized in animal models (30, 31). Recent studies provide multiple novel findings relevant to the development of cell transplantation therapies for treatment of injured or diseased CNS. This study has demonstrated that rhesus mNSCs which are the subpopulations of stem cells present in the adult brain SVZ (32) can survive, differentiate to neurons and promote functional recovery after SCI in addition to other mechanisms which are still unrecognized in rhesus monkeys. Intact axon demyelination and neuronal death are two essential factors attributed to functional loss. Some studies have shown that transplantation of neuronal cells or tissues can cause significant recovery (19). Further studies suggest that embryonic stem cell-derived neurons may be more efficient in functional recovery by re-myelination of axons and compensate for dead neurons (16). However, ethical limitations and lack of accessible sources limit the usage of cells as a powerful treatment for injured organs. For this reason adult stem cells are considered more reliable, accessible sources for cell therapy purposes.

Therefore in the current study mNSCs were isolated from the monkey’s brain (SVZ) as a more suitable source and after characterization were administered to monkeys with contusion SCIs as treatment. We confirmed our isolated mNSCs properties by their abilities to express NSC markers such as nestin, Sox1, and Pax6 (33). Our isolated cell population could differentiate into mature neurons positive for MAP2 (34) (expressed in mature neurons) and GFAP (35) (expressed in glia cells) after at least 35 days of *in vitro* culture. At 6 months after transplantation of isolated mNSCs, we observed a few labeled cells in the spinal cords of the injured animals which differentiated into neural cell types that expressed Tuj1.

There appear to be numerous inflammatory cytokines (36) located in the spinal cord following a contusion injury. According to our results, mNSCs were unable to survive at the injury site for an extended time as evidenced by the few numbers of labeled mature neurons after 6 months. Previous studies have confirmed that NSCs have an immunomodulatory effect (37) which allows for the release of neurotrophin factors and suppressed inflammation at the injury site. Thus, they assist in functional promotion and recovery of injured animals, despite the loss of numerous transplanted cells over time.

On the other hand, the presented behavioral analysis showed significant improvement in the sensory and motor activity of transplanted animals versus the control group. According to the behavioral analysis, tail and hind limb movements were voluntary since the second month after transplantation. In the last month of our follow up, the transplanted animal’s hind limb and tail movement were conscious and showed defensive reflex to the examiners. Their sensory reflexes (sensed pain and pressure) were appropriate and they attempted to avoid the stimulator. Gradual improvement occurred in transplanted and control groups but transplanted animals showed sooner and faster progress than the control group. However, tail and hind limb locomotors and sensory functions improved significantly faster in the transplanted group whereas the control had no improvement until the last week of follow up. In the last week of the study control animals had evidence of perceptible joint movement, while the transplanted groups had active movement and two cases were able to stand but unable to hop. Also, muscle atrophy and bed sores were observed on control animals during the experiment, whereas transplanted monkeys were unaffected by bed sores and atrophic muscles. It seemed that NSCs could protect hind limb muscles and tissues.

Finally, the histological study showed that following trauma to the spinal cord, in some cases, edema and hemorrhage were present. Trauma causes small vacuoles in neural tissue and their combination forms huge vacuoles. Because there is no connective tissue in neural parenchyma, the percentage of fibrosis is the most important factor with which to determine prognosis. Thus, our results have proven this model (according to Fig 1A-D).

According to the histological analysis, we were unable to locate any inflammatory cells such as neutrophils, lymphocytes, plasma cells, and macrophages in any of the tissues. Perhaps prevention of meningeal proliferation can inhibit collagen formation, allowing for a better prognosis. Pathologic cases are classified by the appearance of their features. It is important to note that these are usually present in various combinations in the same case; therefore the "pure" architectural forms of these diagnostic groups may be rare.

In addition, we did not observe any cases of malignancy in any of the injected neural parenchyma or other non-transplanted tissues, including the kidneys, liver, and heart.

These results have suggested that transplanted mNSCs can promote functional recovery in an animal model of contusive SCI. Additional studies are necessary to determine the mechanisms involved in improvement of SCI after cell transplantation.

## Conclusion

Our results indicate that transplantation of neural stem cell-derived adult SVZ in primates can facilitate functional recovery in transplanted animals after a contusion SCI. More importantly, we have not found tumor formation or malignancy in grafted animals even after six months of follow up. Future studies should consider elucidating the mechanisms which involve improvements to sensory motor function in model animals after cell transplantation.
